# Professional Health

**Published:** 1859-12

**Authors:** 


### PROFESSIONAL HEALTH.

The best definition of health will probably be found in "*Harmony of Function.*" To establish anything like a healthy state of the Dental profession, it will be necessary to overhaul the entire old hull; and that task must not be consigned to any other than *competent* hands, if we wish it to *improve* in health; any more than we would call upon a divine to examine and adjust the marred and displaced limbs of the poor victim of some "horrid railroad accident," instead of the cool and well qualified *practical* surgeon.

That this may be well done, it will be necessary to remodel the nomenclature of the profession by such severe pruning, that many of the old fogies will raise their skeleton-like hands over their bald and narrow foreheads in holy horror for the (to them) desecration. A nomenclature, to be useful, must needs be definite; and to be definite and have significance, it must be kept alive by use. To be freely used between the members of the profession, it is of the first importance to be well studied, clearly comprehended, and faithfully improved and recorded.

That it may be recorded, a dictionary of terms must be made, including, of course, many terms not originally, nevertheless strictly dental in their significance, at least in this relation. For all terms that pertain properly to the basal sciences, the culminations of which manifest themselves in dental formulary or modes or means, may be freely and without well-founded objection, pressed into service, with the modifications necessary to make them what it is most desired they should be, clear expositions of the specialities hereunto belonging.

That this may be done in any adequate degree acceptably to those who need it most, it will be necessary to have more than one hand at the work. I would here suggest the propriety of laying the subject before the highest and most harmonious of dental associations, so that their combined intelligence could be called into exercise in selecting and appointing competent men to the various departments, and let them when so appointed, comprise a board of control, before whom the entire work should pass before publication. If this be done, the just complaint of the low state of our profession in a literary point of view, will no longer have a foundation upon which to raise its hateful front.

It may be objected that this is not necessary, in view of the many medical, mechanical, surgical and scientific dictionaries already in the field; to which it is only necessary to appeal for refutation, to the experience of any who have sought out the significations of many terms in use among us; so loosely bandied about that we become doubtful how to define; and after the most thorough search, to our dissatisfaction found so many mutations of signification that *might* be meant, that we were utterly at a loss to decide whether the author or speaker meant this, that or the other.

If no other reason existed for a Dictionary of our *own*, the eminently progressive and developmental character of the speciality would *alone demand* such work.

Dentistry, as a science, sends its roots far into the three kingdoms, from which to draw materials of which to build its beautiful body. And that the suctorial mouths may not be mistaken for those belonging to another department of nature, their specific character must be closely studied and clearly comprehended, which can not be done without the aid of glass and retort—microscope to lay open the minute structures—and alembics to determine proportions. Then with these discoveries for corner-stones, we must institute long, patient and voluminous observations, the work of the many; after which comparisons, make our deductions so as to constitute them demonstrations, thus supplying principles upon which, or in agreement with which we may apply means and modes, constituting the detail of a reliable, satisfactory and eminently successful practice of this culmination of all the merely earthly sciences.

If every step be carefully and well taken, each organ properly conceived and fully developed, all we need is to have each one in normal action to constitute perfect *health* in the professional body.

" Create a soul, and it will aggregate to itself a body suited to its uses," is an aphorism that lies at the very foundation of all physical science. And if the combined efforts of some of the honest, earnest workers in the profession can only be secured, I think SEED may be so fitted by the fulfillment of conditions, as to imprison the form, type, or germinal and developmental power so perfectly as to make it only necesary to bring it into favorable relations, to produce such a well-preportioned and fully grown profession, that it shall be the admiration of every honest beholder; and prove to the world that what is generally called the only legitimate way to generate and develop the professional callings, is no more true than that all the bright children on earth have been begotten and born in legal wedlock. Not to say that the converse is true, which would only enunciate that other anachronism, and say that what is right and proper in municipal relation, can only be proper anywhere in the domain of nature. Nature's laws precede and should underlie all merely municipal regulations—I will not dignify them by saying laws—so long as they are the mere enactments of men, and that not of the best sort.

A certain shrewd and recondite poet said of himself (I will not say whether physically or scholastically),

> " I am the sickly offspring of no foul embrace,
> But stamped in nature's mint of ecstasy;
> I live not to boast, but build a generous race."

And now let each real worker in Dentistry take his cue from this saying, and prove his true legitimacy to the profession of his choice, and do what he can, if not to boast an illustrious line of worthies in this field of usefulness, at least do till death "to build" by his best efforts, a noble and generous platform, upon which a really dignified, because preëminently useful, profession may securely and forever stand. N.

